# The Relationship Between Blood Pressure and Sleep Duration in Turkish Children: A Cross-Sectional Study

**DOI:** 10.4274/jcrpe.4557

**Published:** 2018-02-26

**Authors:** Cengiz Bal, Ahmet Öztürk, Betül Çiçek, Ahmet Özdemir, Gökmen Zararsız, Demet Ünalan, Gözde Ertürk Zararsız, Selçuk Korkmaz, Dinçer Göksülük, Vahap Eldem, Sevda İsmailoğulları, Emine Erdem, Mümtaz M Mazıcıoğlu, Selim Kurtoğlu

**Affiliations:** 1 Eskişehir Osmangazi University Faculty of Medicine, Department of Biostatistics, Eskişehir, Turkey; 2 Erciyes University Faculty of Medicine, Department of Biostatistics; Erciyes Teknopark, Turcosa Analytics Solutions Ltd. Co, Kayseri, Turkey; 3 Erciyes University Faculty of Health Sciences, Department of Nutrition and Dietetics, Kayseri, Turkey; 4 Erciyes University Faculty of Medicine, Department of Pediatrics, Division of Neonatology, Kayseri, Turkey; 5 Erciyes University Faculty of Medicine, Department of Biostatistics; Erciyes Teknopark, Turcosa Analytics Solutions Ltd. Co, Kayseri, Turkey; 6 Erciyes University Halil Bayraktar Health Services Vocational College, Kayseri, Turkey; 7 Trakya University Faculty of Medicine, Department of Biostatistics, Edirne, Turkey; 8 Hacettepe University Faculty of Medicine, Department of Biostatistics, Ankara, Turkey; 9 İstanbul University Faculty of Science, Department of Biology, İstanbul, Turkey; 10 Erciyes University Faculty of Medicine, Department of Neurology, Kayseri, Turkey; 11 Erciyes University Faculty of Health Sciences, Department of Pediatric Nursing, Kayseri, Turkey; 12 Erciyes University Faculty of Medicine, Department of Family Medicine, Kayseri, Turkey; 13 Memorial Hospital, Department of Pediatrics, Kayseri, Turkey

**Keywords:** Adolescent, blood pressure, children, sleep duration

## Abstract

**Objective:**

As in adults, hypertension is also an important risk factor for cardiovascular disease in children. We aimed to evaluate the effect of sleep duration on blood pressure in normal weight Turkish children aged between 11-17 years.

**Methods:**

This cross-sectional study was conducted in the primary and secondary schools of the two central and ten outlying districts of Kayseri, Turkey. Subjects were 2860 children and adolescents (1385 boys, 1475 girls). Systolic and diastolic blood pressures were measured according to the recommendations of the Fourth Report of the National High Blood Pressure Education Program Working Group on High Blood Pressure in Children and Adolescents. Sleep duration was classified as follows: ≤8 hours, 8.1-8.9 hours, 9.0-9.9 hours or ≥10 hours.

**Results:**

For short sleeper boys and girls (participants with a sleep duration ≤8 h) the prevalence of prehypertension and hypertension was 35.0% and 30.8%, respectively. In univariate binary logistic regression analyses (age-adjusted), each unit increment in sleep duration (hours) in boys and girls, decreased the prehypertension and hypertension risk by 0.89 [odds ratio (OR)] [confidance interval (CI); 0.82-0.98] and 0.88 (OR) (CI; 0.81-0.97), respectively (p<0.05). In multiple binary logistic regression analyses [age- and body mass index (BMI)-adjusted] the location of the school and sleep duration categories were shown to be the most important factors for prehypertension and hypertension in both genders, while household income was the most important factor, only in boys.

**Conclusions:**

A sleep duration ≤8 h is an independent risk factor for prehypertension and hypertension in Turkish children aged 11-17 years.

## What is already known on this topic?

Hypertension is an important risk factor for cardiovascular disease in children as well as in adults. Preventive precautions for children and adolescents should be considered in terms of maintaining a healthy lifestyle.

## 

### What this study adds?

A sleep duration ≤8 h is an independent risk factor for prehypertension and hypertension in Turkish children aged 11-17 years.

## Introduction

In recent years, the prevalence of both hypertension and prehypertension are increasing worldwide (1,2,3). Increased levels of high blood pressure (BP) in childhood is an indicator for an increase in prevalence of coronary artery disease in adulthood. Sedentary lifestyle, obesity and nutritional habits are modifiable risk factors known to effect development of hypertension in childhood. The relationship between sleep duration and BP is an issue for both experimental and epidemiological studies. Both hypertension and coronary artery disease have been associated with sleep duration in adults (4,5). However, the etiology of this relationship is not fully understood. Changes in hormonal activity, increased activity of the renin-angiotensin-aldosterone system and changes in circadian rhythm have been reported to have a role in this relationship (6,7,8,9,10). 

The best approach to prevent hypertension-related mortality or morbidity is to prevent and treat the hypertension (11). However the treatment of hypertension is difficult and complex. Despite major advances in treatment of hypertension in the past few decades, the majority of patients fail to achieve treatment goals. Lifestyle changes including a low salt diet, weight loss, regular physical activity, cessation of smoking and diet therapy are important in decreasing the risk of hypertension-related disease (12,13). 

Much current epidemiologic research, related to lifestyle, focuses on the relationship between short sleep duration and hypertension prevalence (11,13). In the current study, we aimed to evaluate the effects of lifestyle characteristics on BP among children living in one of the biggest city in Central Anatolia, Kayseri.

## Methods

This cross-sectional study was performed on a large cohort of children, aged 11-17 years. All children attended primary and secondary schools located centrally (two schools) and in the outlying districts (ten schools) of the town of Kayseri in Turkey. Kayseri is one of the largest three cities in Central Anatolia with more than 1.000.000 inhabitants. Data were obtained from the “Determination of Anthropometric Measurements of Turkish Children and Adolescents” survey (14,15).

The study was approved by the Ethics Committee of Erciyes University School of Medicine and Kayseri Province Educational Board (approval number: 04/312, approval date: 07/09/2004). Written consent was taken from the parents at the beginning of the study in accordance with the Declaration of Helsinki. Decimal age was calculated by subtracting the birth date from the observation date (16).

### Anthropometric Indices

All anthropometric indices were measured twice by experienced health professionals and the mean value was recorded for analyses. All inter-observer correlation coefficients were ≥0.91.

Weight was measured using a standard beam balance sensitive to 0.1 kg, using a Tefal Ultraslim (France), in minimal clothing (bare feet and with light clothing).

Height was measured with a portable Seca stadiometer with a sensitivity of 0.1 cm. Daily calibration was made to the device. Measurements were made with the subject barefoot, the heels, hip and shoulders touching the stadiometer and the head in neutral position with eyes gazing forward. 

Body mass index (BMI) was calculated by dividing body weight in kilograms by the square of body height in meters (kg/m^2^).

Systolic BP (SBP), and diastolic BP (DBP) were measured according to the recommendations of the Fourth Report of the National High Blood Pressure Education Program Working Group on High Blood Pressure in Children and Adolescents (15,17). According to BP charts for age, gender and height, normal, pre- and hypertension were defined as SBP and DBP below the 90^th^ percentile, between 90^th^-<95^th^ percentile and equal to or higher than 95^th^ percentile respectively (15).

Several anthropometric indices were measured: waist circumference (WC), mid-upper arm circumference (MUAC), triceps skinfold thickness, arm fat area, MUAC fat percentage (14). WC was divided by height to yield the WC to height ratio.

### Demographics

A questionnaire form constructed by the researchers with socio-demographic variables was used. Computer use (hours), sleep duration (hours), TV viewing (hours), location of residence (urban/suburban), mode of transport to school (walking or by car), self-reported household income (good, fair, poor), house size (m^2^), parental education, elevator use (none/habitual), maternal employment (housewife/employed) were asked (14).

The questionnaire was sent to parents and the question related to sleep was asked as, “How many hours of sleep does your child usually get?”, aiming to provide information to the researchers on the child’s habitual sleep duration. Sleep duration was classified into four groups as: ≤8 h, 8-9 h, 9-10 h, and ≥10 h and these categories were selected to compare the results of the current study with similar studies (18).

The following sociodemographic and behavioral effectors of obesity were analysed: computer use, sleep duration, viewing duration of TV, place of residence, appetite, mode of transport to school, household income, house size, parental education, maternal employment and elevator use (14).

### Statistical Analysis

Chi-square tests were used to determine significant differences in proportions among categorical variables and to compare continuous variables independent sample’s t-test was used. Anthropometric indices were compared with sleep duration categories by analysis of covariance (adjustment for multiple comparisons: Bonferroni), where age or age and BMI were the covariates. Univariate (adjusted age) and multiple (the backward stepwise procedure; adjusted age or age and BMI) binary logistic regression analyses were used to examine the risk factors to influence whether SBP or DBP≥90^th^ percentile (prehypertension and hypertension). All statistical analyses were performed by R2.14.0 program (www.r-project.org). Two-tailed p-values of <0.05 were considered to be statistically significant.

## Results

The study included 2860 children. Of the sample 1385 (48.4%) were boys and 1475 (49.6%) were girls. Prehypertension and hypertension together (DBP≥90^th^) was found in 485/1385 (35.0%; 95% CI 0.32.5-0.37.6) of boys and 455/1475 (30.8%; 95% CI 0.28.5-0.33.3) of girls, respectively. Prehypertension prevalences in this group were 26.3% and 22.4% for boys and girls, respectively. In both genders, we found that increased sleep duration was significantly related with decreased prehypertension and hypertension (Table 1). In Table 2, the relationship between sleep duration (≤8 h, 8.1-8.9 h, 9.0-9.9 h, ≥10 h), anthropometric indicators of metabolic risk and BP is shown. The unique significant finding in this comparison was the reduction in mean SBP from 112.9 mmHg to 107.9 mmHg in girls and the reduction in mean DBP from 72.7 mmHg to 69.4 mmHg in boys as sleep duration increases.

In Tables 3A and 3B, the effects of prehypertension on sociodemographic and behavioural variables for children and adolescents (by univariate and multiple binary logistic regression analyses) are shown. Increase in sleep duration and decrease in BMI are two indicators of normal BP in both genders. The corresponding odds for sleep duration were OR: 0.89, CI; 0.82-0.98 and OR: 0.88, CI; 0.81-0.97, respectively for boys and girls. The corresponding odds for BMI were OR: 1.12, CI; 1.08-1.16 and OR: 1.09, CI; 1.06-1.14 respectively for boys and girls (Table 3A).

We adjusted our dependent variables (normotensive/prehypertensive and hypertensive) for BMI and age in multiple binary logistic regression. Independent variables in this categoric analysis that were statistically significant (p<0.05) were; location of the school (in both genders), sleep duration (in both genders) and household income (only in boys). There was no relationship between maternal employment, paternal education, house size, elevator use and mode of transport to school (p>0.05). Sleeping 8-9 hours compared to sleeping less than 8 hours in boys and sleeping more than 10 hours compared to sleeping less than 8 hours a day in girls were the risk factors for increased BP. Residing in urban versus rural areas was shown to be a risk factor for increased prehypertension and hypertension risk; OR: 1.43, CI: 1.11-1.84, OR: 1.77; CI; 1.37-2.27, respectively in boys and girls (Table 3B, p<0.05). 

**Figure 1:**
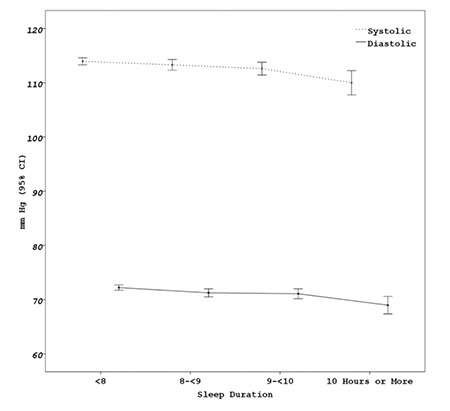
Systolic and diastolic blood pressure values according to sleep duration

**Figure 2:**
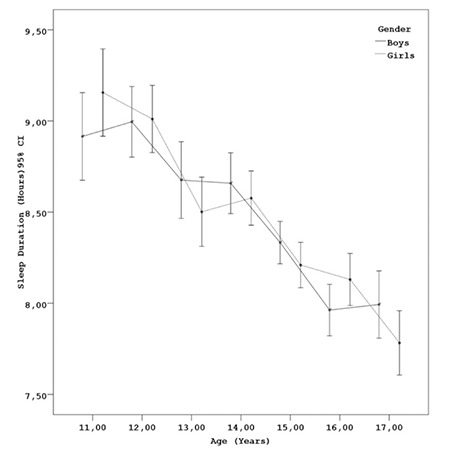
The distribution of sleep duration by age groups in boys and girls

SBP and DBP values according to sleep duration are shown in Figure 1. Longer sleep duration was associated with lower BP. The distribution of sleep duration for age groups in boys and girls is given in Figure 2. In both genders, sleep duration showed a decrease with increase in age.

## Discussion

Previous studies on adults indicate that there is a strong relationship between sleep duration and increased BP (19,20,21). To the best of our knowledge, the current study is the first to demonstrate a relationship between short sleep duration and high BP in children and adolescents aged 11-17 years. According to the univariate binary logistic regression analyses, sleep duration less than 8 h is a significant risk factor for hypertension in both genders. With each increment in sleeping hours for boys and girls, the risk of increased BP decreases (OR:0.89, CI: 0.82-0.98 and OR: 0.88, CI: 0.81-0.97 respectively in boys and girls). In multiple binary logistic regression analyses (adjusted for age and BMI) and by categorising sleep duration into four groups (≤8 h, 8.1-8.9 h, 9.0-9.9 h, ≥10 h), shorter sleep duration was also shown to be a risk factor for increased BP. There are several studies examining the relationship between increased BP and various environmental conditions, other than sleep duration (22,23,24,25,26). In this present study, the only other significant environmental risk factor identified was location of the school the children or adolescents were attending and this was only true of the girls. No other environmental risk factors included in this study were found to be an independent risk factor in our data set.

The American Academy of Sleep Medicine developed new consensus recommendations for the amount of sleep needed to promote optimal health, including avoidance of hypertension in children and adolescents. According to these recommendations, teenagers 13-to-18 years of age should sleep 8-to-10 hours per 24 hours on a regular basis to promote optimal health (27). 

In a recent review on sleep characteristics and cardiovascular risk in children and adolescents, sleep pattern is indicated as a significant risk factor for cardiovascular disease, however the sleep pattern may differ between different geographic locations (28). Similarly, Meininger et al (29), demonstrated that long sleep duration is associated with a decrease both in SBP and DBP. In the study by Archbold et al (30) in 334 children aged 6-11 years, both a decrease in sleep duration and an increase in BMI were associated with increased BP. Prehypertension and hypertension were both associated with short sleep duration (less than eight hours) among Lithuanian children and adolescents (n=6940) aged 12 to 15 years; when adjusted for age, gender, BMI, physical activity and smoking (31).

However, a recent longitudinal analysis from early-to-late adolescence found no association between sleep duration and BP in females. However, in males longer sleep duration was associated with lower values of BP (32).

Our study was conducted in a large province of central Anatolia in Turkey. In other previous studies, higher salivary cortisol levels were detected in individuals with short sleep when compared with normal or high sleep duration (33). In healthy young adults, impaired vascular endothelial adhesion markers and also endothelial-dependent/independent microvascular reactivity were detected in acute total sleep deprivation (34). Additionally, decreased endothelial-dependent vasodilatation, representing impaired endothelial function, was reported in participants with short sleep duration. In another study, participants with a sleep duration of less than five hours were found to have significant changes in heart rates and BP values (35). Significant increases in catecholamine levels measured in 24-hour urine samples were reported in adults, suggesting an increase in sympathetic activity related to short sleeping hours (36).

In accordance with previous studies, we found a relationship between short sleep duration and hypertension in children and adolescents of both genders (32,33). In the current study, with the decrement in sleep duration, hypertension risk is increased. However, in previous studies, the relationship betwen short sleep duration and the risk of hypertension in adult women were explained by structural characteristics (BMI and other body composition variables) and menopause (37,38,39).

Peach et al (40), examined BMI as a possible mediator of the effect of sleep duration on risk for hypertension in a sample of sixth graders. Among boys, all three sleep characteristics (school-night sleep duration, weekend night sleep duration, and daytime sleepiness) predicted BMI and yielded significant indirect effects on risk for hypertension. In girls on the other hand, only daytime sleepiness predicted BMI and yielded a significant indirect effect on risk for hypertension.

The primary contribution of the current study may be revealing the age- and BMI-adjusted risk factors for increased BP associated with decreased sleeping duration.

### Study Limitations

However, the main limitation of our study is that we did not use a validated scale to assess sleep duration and relied on self-reports of the parents for sleep duration of their children (41,42,43,44,45,46,47,48). The cross-sectional measurement of BP to assess increased BP is also a limitation of this study.

## Conclusion

To the best of our knowledge, the current study is the the first to demonstrate that short sleep duration is a risk factor for increased BP in non-obese, Turkish children. We believe this issue requires further exploration. 

## Figures and Tables

**Table 1:**
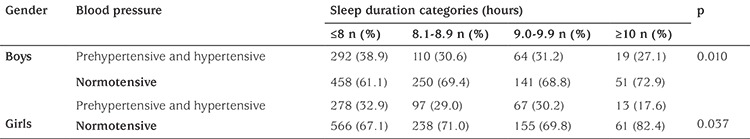
The relationship between sleep duration and blood pressure for boys and girls

**Table 2:**
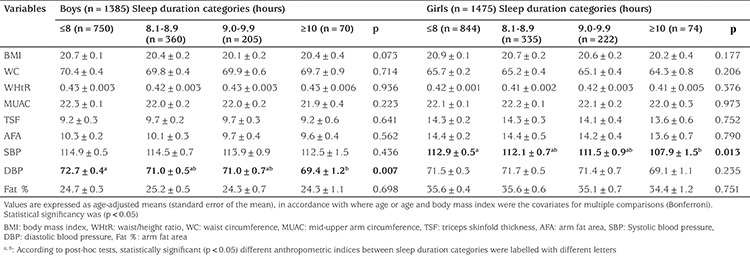
The comparison of several variables by sleep duration categories in boys and girls

**Table 3A:**
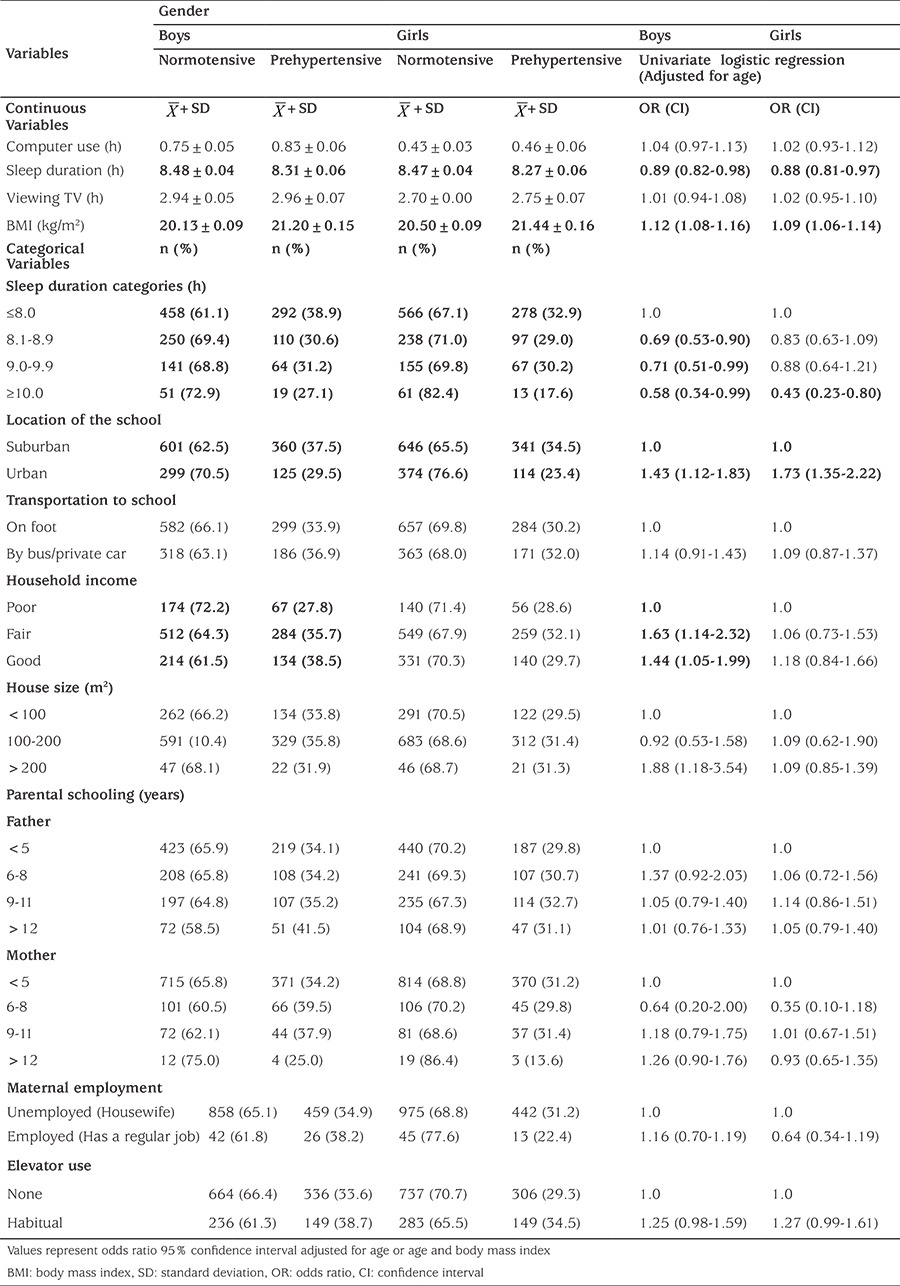
Univariate and multiple binary logistic regression of the likelihood of prehypertension on sociodemographic and behavioural variables for children and adolescents [with odds ratio and 95% confidence intervals]

**Table 3B:**
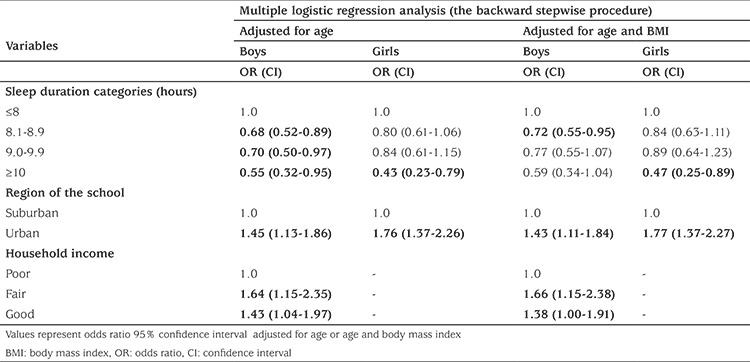
Multiple logistic regression analysis of the likelihood of prehypertension on sociodemographic and behavioural variables for children and adolescents
